# Genomic profiling of multiple breast cancer reveals inter-lesional heterogeneity

**DOI:** 10.1038/s41416-019-0713-1

**Published:** 2020-01-13

**Authors:** Soomin Ahn, Hyun Jeong Kim, Eunyoung Kang, Eun-Kyu Kim, Se Hyun Kim, Jee Hyun Kim, In Ah Kim, So Yeon Park

**Affiliations:** 1 Department of Pathology, Seoul National University Bundang Hospital, Seoul National University College of Medicine, Seongnam, Gyeonggi Republic of Korea; 2 Department of Surgery, Seoul National University Bundang Hospital, Seoul National University College of Medicine, Seongnam, Gyeonggi Republic of Korea; 3 Division of Hematology and Medical Oncology, Department of Internal Medicine, Seoul National University Bundang Hospital, Seoul National University College of Medicine, Seongnam, Gyeonggi Republic of Korea; 4 Department of Radiation Oncology, Seoul National University Bundang Hospital, Seoul National University College of Medicine, Seongnam, Gyeonggi Republic of Korea

**Keywords:** Breast cancer, Cancer genetics

## Abstract

**Background:**

Multiplicity in breast cancer is common. Studies on multiple breast cancers have revealed high concordance in biomarker status among individual lesions. However, genomic differences among multiple lesions are not well-established. We aimed to investigate the potential genomic heterogeneity of multiple breast cancer.

**Methods:**

Twenty-one patients with radiologically and histologically evident multiple breast cancer with similar histology were included. Two lesions from each of the 21 patients were selected, and biomarker status was evaluated for each lesion. Capture-based targeted next-generation sequencing was performed using a cancer gene panel consisting of 170 genes.

**Results:**

We identified discordance in intrinsic subtype in 2 (10%) of the 21 patients. Pathogenic mutations were detected in 13 of the 21 patients, of whom 11 shared oncogenic variants in the two lesions. The remaining two patients yielded different mutation results for *TP53*, *ATM*, and *PIK3CA*. Difference in copy number alteration was observed in 7 (33%) of the 21 patients including *ERBB2* (*n* = 2), *FGFR1* (*n* = 2), and *FGFR2* (*n* = 1) genes.

**Conclusion:**

Despite similar histologic features of the individual lesions, inter-lesional genomic difference was identified in more than one-third of the patients. Inter-lesional genomic heterogeneity needs to be considered when performing a genomic test in multiple breast cancers.

## Background

Multiple (multifocal or multicentric) breast cancers are a relatively frequent entity with a reported incidence ranging from 6 to 60% in the literature.^1–5^ A multifocal breast cancer usually refers to two or more separate tumours located in the same quadrant while a multicentric breast cancer denotes to two or more separate tumours occupying more than one quadrant of the same breast.^5^ However, there exists no international consensus on the definition of multifocality or multicentricity, and the distinction is often difficult. Multiple breast cancers are associated with increased regional lymph node metastasis as well as unfavourable prognosis compared to unifocal breast cancers.^3,4^ Considering the more aggressive biologic behaviour of multiple breast cancer, it is recommended to document the multiplicity by placing the (m) modifier for the T category in the current American Joint Committee on Cancer manual.^6^ Since multiple breast cancers are known to share similar histologic features and biomarker status,^7,8^ current guidelines allow performing oestrogen receptor (ER), progesterone receptor (PR), and human epidermal growth factor receptor 2 (HER2) immunohistochemistry (IHC) in the largest tumour as representative of the multiple tumours.^9,10^

Breast cancer is a genetically heterogeneous disease,^11^ and recent advances in single-cell sequencing and liquid biopsy have provided insight into genomic heterogeneity among sub-clonal tumour cell populations during disease progression.^12,13^ However, genomic differences among multiple lesions are not well-established. A recent study reported that genomic heterogeneity was common among different lesions of multiple breast cancer.^14^

In the era of precision medicine, the number of molecular alterations possessing potential clinical utility is steeply rising; a genomic test using the next-generation sequencing (NGS) technique can be rapidly applied in the clinic. Adequate sampling of a tumour is a prerequisite for genomic testing, and it is usually retrieved from a metastatic tumour to account for genetic evolution during metastatic progression. However, in patients with metastatic breast cancer at inaccessible sites, it is inevitable to perform a genomic test on the primary tumour. In multiple breast cancers, inter-lesional genomic heterogeneity can complicate treatment decisions based on genomic information.^15^ In this study, we investigated inter-lesional genomic differences in multiple breast cancers with similar histology to provide a guide on genomic testing in multiple breast cancer.

## Methods

### Patient selection and data collection

Patients with primary multiple breast cancer who received surgery at Seoul National University Bundang Hospital between 2009 and 2012 were included. The inclusion criteria were as follows: (1) no neoadjuvant chemotherapy, (2) grossly and radiologically separate lesions, (3) histologically confirmed separate lesions with at least 5 mm interval in between, (4) size of individual lesions at least 5 mm, and (5) same histology and grade. The distinction between multifocal and multicentric breast cancers was impracticable. Twenty-one patients with multiple breast cancer who met the inclusion criteria were finally chosen for this study. We selected two lesions (tumours #1 and #2) from each patient after reviewing the haematoxylin and eosin (H&E)-stained slides. Tumour #1 represented the largest tumour, and tumour #2 represented the second-largest tumour. We collected the following data: size of each tumour, number of individual tumours, distance between the tumours, lymph node status, histologic grade, presence of ductal carcinoma in situ, extensive intraductal component, and lymphovascular invasion after reviewing the pathology reports and H&E-stained slides. This study was approved by the Institutional Review Board (IRB) at Seoul National University Bundang Hospital (IRB No. B-1902-522-301). Informed consent was waived since this study was a retrospective study using archival paraffin blocks and the samples were anonymised for this study.

### Immunohistochemistry and definition of breast cancer subtypes

All of the 21 patients had standard biomarker information including ER, PR, and HER2 status, and Ki-67 for the largest-index tumour (tumour #1); however, IHC had not been performed for tumour #2. Thus, we used the following antibodies that had been used for evaluation of tumour #1 and performed IHC for tumour #2: ER (1:100; clone SP1; Labvision, Fremont, CA), PR (1:70; PgR 636; Dako, Carpinteria, CA), HER2 (ready to use; 4B5; Ventana Medical Systems, Tucson, AZ), and Ki-67 (1:250; MIB-1; Dako). Immunohistochemical staining on representative tissue sections was carried out in a BenchMark XT autostainer (Ventana Medical Systems) using an UltraView detection kit (Ventana Medical Systems).

Immunohistochemical expression of the standard biomarkers was used to categorise the tumour samples into breast cancer subtypes according to the 2011 St. Gallen Expert Consensus as follows^16^: luminal A (ER+ and/or PR+, HER2−, Ki-67 <14%), luminal B/HER2-negative (ER+ and/or PR+, HER2−, Ki-67 ≥14%), luminal B/HER2-positive (ER+ and/or PR+, HER2+), HER2-positive (ER−, PR−, HER2+), and triple-negative subtype (ER−, PR−, HER2−). For ER and PR, 1% or greater nuclear staining in tumour cells was considered positive. For HER2, 3+ on IHC or the presence of gene amplification on in situ hybridisation was considered positive.

### DNA library preparation and next-generation sequencing

Deep targeted DNA sequencing was performed using a cancer-related gene panel consisting of 170 widely known cancer-associated genes (Supplementary Table S1). Tumour-rich areas were marked for manual macro-dissection. The tumour purity ranged from 50 to 90% (Supplementary Table S2). Genomic DNA was extracted from each of the formalin-fixed, paraffin-embedded tissue samples using the QIAamp® DNA FFPE Tissue kit (Qiagen, Hilden, Germany), and 200 ng of DNA was used for library generation. DNA library preparation and target enrichment by the hybrid capture method were performed according to Illumina’s standard protocol using Agilent SureSelectXT Target Enrichment Kit (Agilent Technologies, Santa Clara, CA). Target region bases were sequenced for each sample using the HiSeq 2500 system (Illumina, San Diego, CA), achieving an average coverage depth ×715 (Macrogen Inc., Seoul, Republic of Korea).

### Sequence alignment and variant calling

Adapter sequences were removed from the raw sequencing reads by cutadapt.^17^ Trimmed reads were aligned to the reference genome (GRCh37/hg19) using Burrows−Wheeler Aligner-MEM (BWA-MEM).^18^ Poorly mapped reads with a mapping quality (MAPQ) below 20 were removed using Samtools version 1.3.1.^19^ Somatic mutations including single nucleotide variants (SNV) and small insertions and deletions (INDELs) were detected by the MuTect2 algorithm.^20^ All the variants were annotated using SnpEff & SnpSift v4.3i^21^ with dbNSFP v2.9.3.^22^

### Single nucleotide variant and copy number analysis

Since matched germline samples were not available, we made efforts to reduce the effect of false-positive variants and germline contamination. The following criteria were used to filter out the less significant variants and narrow down to the clinically relevant variants: (i) variants <3% allele frequency and <100× read depth at the variant were excluded; (ii) variants with an allele frequency greater than 0.1% in the Exome Aggregation Consortium (ExAC) East Asian database were excluded;^23^ (iii) all synonymous, intronic, 30- and 50 untranslated region (UTR) variants were excluded, and (iv) variants previously reported to be benign or likely benign in the ClinVar (2017-06 release) archive^24^ were excluded. Finally, pathogenic mutations were selected referring to COSMIC.^25^ We performed copy number alteration analysis using targeted sequencing data through methods developed in-house that were adapted and modified from CNVkit package.^26^ Copy number variation (CNV) data were exported as log 2 ratio values. In case of log 2 ratio > 0.5 of *FGFR1*, *FGFR2*, and *ERBB2* genes, in situ hybridisation was performed. In other genes, log 2 ratio > 1 (estimated gene copies > 4) was regarded amplification.

### Sanger sequencing

We performed Sanger sequencing to confirm point mutations (*PIK3CA* E726K, *ATM* R1466*, and *TP53* H179Y) that were found to be discordant in the two lesions from two patients. Fifty nanograms of DNA was amplified in a 20 μL reaction volume containing AccuPower® ProFi Taq PCR PreMix (Bioneer, Daejeon, South Korea). Primers were designed using the Primer Design Tool from NCBI. The primers were as follows: *PIK3CA* exon 14 forward 5′-CCT GAA ACT CAT GGT GGT TTT-3′, *PIK3CA* exon 14 reverse 5′-GCT GAG AGG CAG TGG AAC TT-3′, *ATM* exon 29 forward 5′-TCA AAC CCA AAT CTA AAT TCT GTT A-3′, *ATM* exon 29 reverse 5′-TCA AAC CCA AAT CTA AAT TCT GTT A-3′, *TP53* exon 5 forward 5′-GTT TCT TTG CTG CCG TCT TC-3′, *TP53* exon 5 reverse 5′-ACA CGC AAA TTT CCT TCC AC-3′. The sequencing reactions were loaded on 3730xl DNA Analyzer from Hitachi (Applied Biosystems, Foster City, CA). Sequence traces from the tumour DNA samples were aligned with the genomic reference sequence and analysed using SeqPilot software (Applied Biosystems).

### Silver in situ hybridisation

HER2 silver in situ hybridisation (SISH) assays were performed with INFORM HER2 DNA and Chromosome 17 probes (Ventana Medical Systems) using an ultraView SISH Detection Kit (Ventana Medical Systems) as previously described.^27^ After scanning the whole section, at least 50 cells were evaluated in each case, and HER2 status was determined according to the updated 2013 ASCO/CAP guidelines.^28^ HER2-equivocal cases were regarded as HER2 non-amplified.

### Florescence in situ hybridisation

Four-micrometer deparaffinised section was incubated in pre-treatment solution (Abbott Molecular, Downers Grove, IL) at 80 °C for 40 min and then in protease solution (Abbott Molecular) for 40 min at 37 °C. Fibroblast growth factor receptor 1 (*FGFR1*) FISH was performed with locus-specific bacterial artificial chromosome (BAC), RP11-100B16 (chr8:38,358,839-38,522,417). We obtained the BAC clone from Invitrogen (Carlsbad, CA) and purified it with a large construction kit (Qiagen, Valencia, CA). DNA from the BAC clone was labelled with SpectrumOrange using a nick translation kit (Abbott Molecular). *FGFR1* BAC probe and *FGFR2* probe (Vysis LSI FGFR2 SpectrumOrange Probe, 08N42-020, Abbott Molecular) were denatured at 73 °C for 5 min and hybridised at 37 °C for 20 h. Post-hybridisation washes were performed according to the protocol supplemented. Slides were mounted in 4′,6-diamidino-2-phenylindole/anti-fade and viewed with a fluorescence microscope. The *FGFR1 or FGFR2* was considered to be amplified if the average gene copy number was ≥6, and high-level amplification was defined as an average gene copy number ≥10.

## Results

### Patient demographics and clinicopathologic features

Clinicopathologic characteristics of the 21 patients are summarised in Table 1. The pathologic T stage was pT1 in 10 (48%) patients, pT2 in 9 (43%) patients, and pT3 in 2 (10%) patients. Of the total 21 patients, 9 (43%) had lymph node metastasis. All patients had invasive breast cancer of no special type. Histologic grade was I for 1 (5%), II for 6 (29%), and III for 14 (67%) patients. Ductal carcinoma in situ was identified in 20 (95%) of 21 patients, and an extensive intraductal component was present in 4 (19%) patients. Lymphovascular invasion was identified in 12 (57%) patients. For the index tumour (tumour #1), ER and PR were positive in 15 (71%) and 14 (67%) cases, respectively. HER2 IHC was negative (0 or 1+) in ten (48%) cases, equivocal (2+) in six (29%) cases, and positive (3+) in five (24%) cases. Of the HER2 IHC-equivocal cases, three showed *ERBB2* amplification on SISH. The distance between tumours #1 and #2 ranged from 5 to 45 mm with an average of 17 mm. The number of multiple lesions was 2 in 13 (62%) patients and 3 or more in 8 (38%) patients. Of the 21 patients, one patient (patient 2) developed distant metastasis to the lung and brain and died of breast cancer.

**Table 1:** Patients characteristics.

Variables	No. of patients	%
pT stage		
pT1	10	47.6
pT2	9	42.9
pT3	2	9.5
pN stage		
pN0	12	57.1
pN1	6	28.6
pN2	1	4.8
pN3	2	9.5
Histologic grade		
I	1	4.8
II	6	28.6
III	14	66.7
Ductal carcinoma in situ		
Absent	1	4.8
Present	20	95.2
Extensive intraductal component		
Absent	17	81.0
Present	4	19.0
Lymphovascular invasion		
Absent	9	42.9
Present	12	57.1
Oestrogen receptor		
Positive	15	71.4
Negative	6	28.6
Progesterone receptor		
Positive	14	66.7
Negative	7	33.3
HER2^a^		
Negative	13	61.9
Positive	8	38.1
Ki-67		
<20%	9	42.9
≥20%	12	57.1
Subtype^a^		
Luminal A	5	23.8
Luminal B/HER2 negative	7	33.3
Luminal B/HER2 positive	3	14.3
HER2 positive	5	23.8
Triple-negative	1	4.8
Distance between lesions (mm)		
≤10	11	52.4
>10	10	47.6
Number of lesions		
2	13	61.9
≥3	8	38.1
Recurrence		
No	20	95.2
Yes	1	4.8
Death		
Alive	20	95.2
Dead	1	4.8

^a^In index tumour

### Comparison of intrinsic subtype in two lesions

**Fig. 1:**
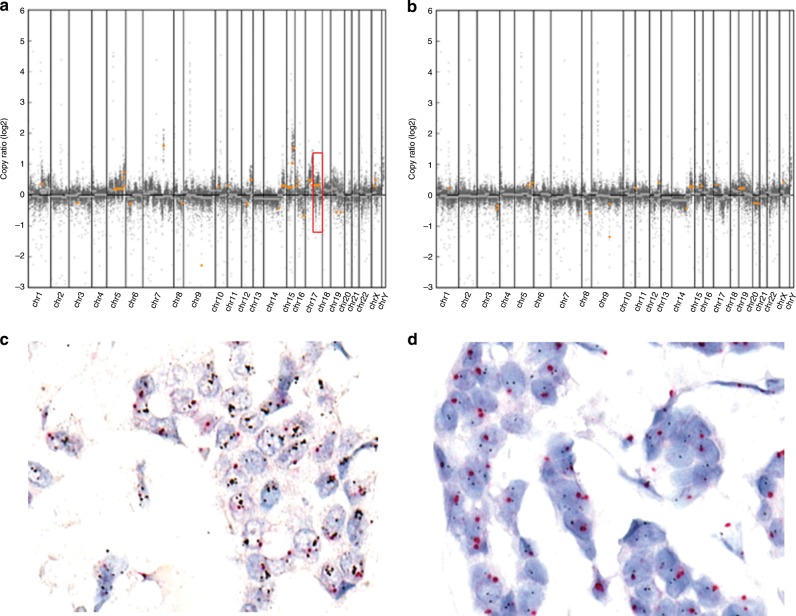
A representative case with inter-lesional copy number heterogeneity in *ERBB2* gene. Genome-wide copy number frequency plots (**a** Tumour #1, **b** Tumour #2) and *ERBB2* silver in situ hybridisation (SISH) images (**c** Tumour #1, **d** Tumour #2) in patient 9. Despite similar histologic features and identical HER2 immunohistochemistry results (2+), *ERBB2* amplification is identified only in tumour #1 (box). SISH confirms amplification with average *ERBB2* copy number of 5.7 in tumour #1. In addition to *ERBB2*, *SMO*, *NTRK3*, *IDH2*, *IGF1R*, and *CDK12* amplifications are also detected in tumour #1.

In the index tumour, the number of luminal A, luminal B/HER2-negative, luminal B/HER2-positive, HER2-positive, and triple-negative subtypes was 5 (24%), 7 (33%), 3 (14%), 5 (24%), and 1 (5%), respectively. Except for two patients, 19 (91%) patients shared the same subtype in tumours #1 and #2. In case of patient 19, tumour #1 was HER2-positive, and tumour #2 was triple-negative. In patient 9, tumour #1 and tumour #2 had the same IHC results (ER+, PR+, and HER2 2+). Tumour #1 had low-level *ERBB2* amplification; however, tumour #2 showed no *ERBB2* amplification on HER2 SISH during the study. Thus, tumour #1 was classified as luminal B/HER2-positive and tumour #2 as luminal B/HER2-negative (Fig. 1).

### Comparison of point mutations in two lesions

Table 2 summarises the mutations detected. The most frequent pathogenic mutation belonged to *TP53* (38%) followed by *PIK3CA* (29%). Pathogenic mutations were detected in 13 of 21 patients, of whom 11 (85%) harboured the same mutations in both lesions. The remaining two patients had different results for mutation in *PIK3CA* and *ATM* (patient 3), and *TP53* (patient 8). In patient 3, a *PIK3CA* variant, p.E726K, was detected only in tumour #2, and the variant allele frequency was 14%. An *ATM* variant, p.R1466*, was also detected only in tumour #2, and the variant allele frequency was 3%. In patient 8, a *TP53* variant, p.H179Y, was detected only in tumour #2, and the variant allele frequency was 3%.

**Table 2:** Detected pathogenic point mutations.

	Tumour 1		Tumour 2	
Patient 1				
Patient 2				
Patient 3			***PIK3CA***	**p.E726K**
	*PIK3CA*	p.H1047R	*PIK3CA*	p.H1047R
			***ATM***	p.R1466*
Patient 4	*PIK3CA*	p.H1047L	*PIK3CA*	p.H1047L
Patient 5	*AKT1*	p.E17K	*AKT1*	p.E17K
Patient 6				
Patient 7				
Patient 8	*TP53*	p.P146S	*TP53*	p.P146S
			***TP53***	**p.H179Y**
Patient 9				
Patient 10	*PIK3CA*	p.E542K	*PIK3CA*	p.E542K
	*TP53*	p.Y104C	*TP53*	p.Y104C
Patient 11				
Patient 12	*PIK3CA*	p.H1047R	*PIK3CA*	p.H1047R
Patient 13	*CDH1*	p.V832M	*CDH1*	p.V832M
	*TP53*	p.G266V	*TP53*	p.G266V
Patient 14				
Patient 15				
Patient 16	*AKT1*	p.E17K	*AKT1*	p.E17K
	*TP53*	p.H179Y	*TP53*	p.H179Y
Patient 17	*TP53*	p.R248W	*TP53*	p.R248W
Patient 18	*TP53*	p.Y220C	*TP53*	p.Y220C
Patient 19				
Patient 20	*PIK3CA*	p.H1047R	*PIK3CA*	p.H1047R
	*PTEN*	p.Q214*	*PTEN*	p.Q214*
	*PTEN*	p.Q245*	*PTEN*	p.Q245*
	*TP53*	p.V274A	*TP53*	p.V274A
Patient 21	*PIK3CA*	p.H1047R	*PIK3CA*	p.H1047R
	*TP53*	p.S127F	*TP53*	p.S127F

The discordant results between tumour #1 and #2 are marked with bold letters

The distance between the tumour pairs showing inter-lesional mutation heterogeneity was 3.5 and 4 cm. Although we did not perform statistical analysis due to the small number of cases, these two tumour pairs tended to have a greater inter-lesional distance compared to the other tumour pairs. The inter-lesional distances for 21 cases are shown in Supplementary Table S2.

We performed Sanger sequencing to confirm the discordant point mutations (*PIK3CA*, *ATM*, and *TP53*) in each lesion of patients 3 and 8. However, we failed to confirm these mutations on Sanger sequencing probably due to their low mutation frequencies.

### Comparison of copy number alterations in two lesions

The copy number alterations in the 21 patients are summarised in Table 3. Copy number alteration was detected in 12 patients.

**Table 3:** Detected copy number alterations.

	Tumour 1	Tumour 2
Patient 1	***CCND1***	
Patient 2		
Patient 3	***FGFR1***	
Patient 4	*PIK3CA*	*PIK3CA*, ***FGFR2***
Patient 5		
Patient 6		
Patient 7	*AKT1*	*AKT1*
Patient 8	*ERBB2*	*ERBB2*
Patient 9	***ERBB2, SMO, NTRK3, IDH2, IGF1R, CDK12***	
Patient 10	***CCND1, TSC2***	
Patient 11		
Patient 12		
Patient 13	*CDK12*, *ERBB2*	*CDK12*, *ERBB2*, ***FGFR1***
Patient 14		
Patient 15	*CDK12*, *ERBB2*	*CDK12*, *ERBB2*
Patient 16		
Patient 17	*ERBB2*	*ERBB2*
Patient 18	*CDK12*, *ERBB2*	*CDK12*, *ERBB2*
Patient 19	***ERBB2***	
Patient 20		
Patient 21		

The discordant results between tumour #1 and #2 are marked with bold letters

**Fig. 2:**
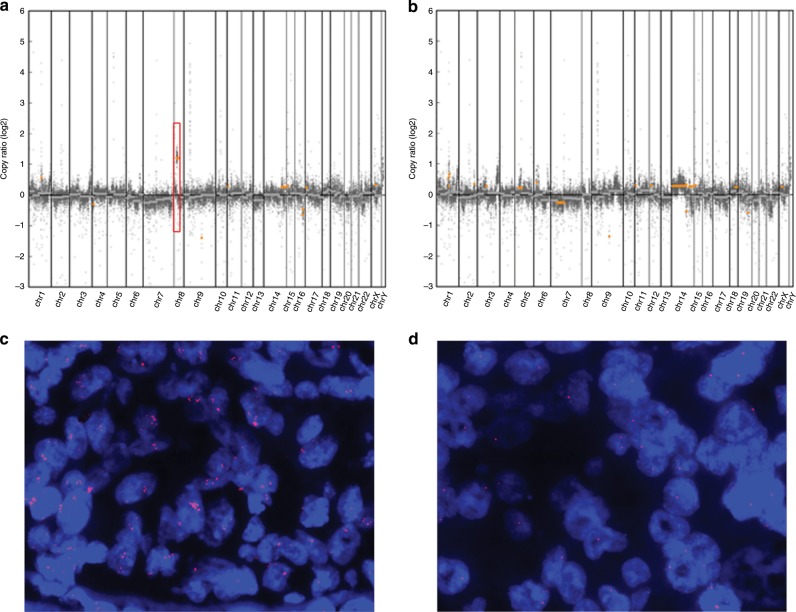
A representative case with inter-lesional copy number heterogeneity in *FGFR1* gene. Genome-wide copy number frequency plots (**a** Tumour #1, **b** Tumour #2) and *FGFR1* florescence in situ hybridisation (FISH) images (**c** Tumour #1, **d** Tumour #2) in patient 3. *FGFR1* amplification is detected only in tumour #1 (box). Average copy number of *FGFR1* is 9.1 on FISH.

**Fig. 3:**
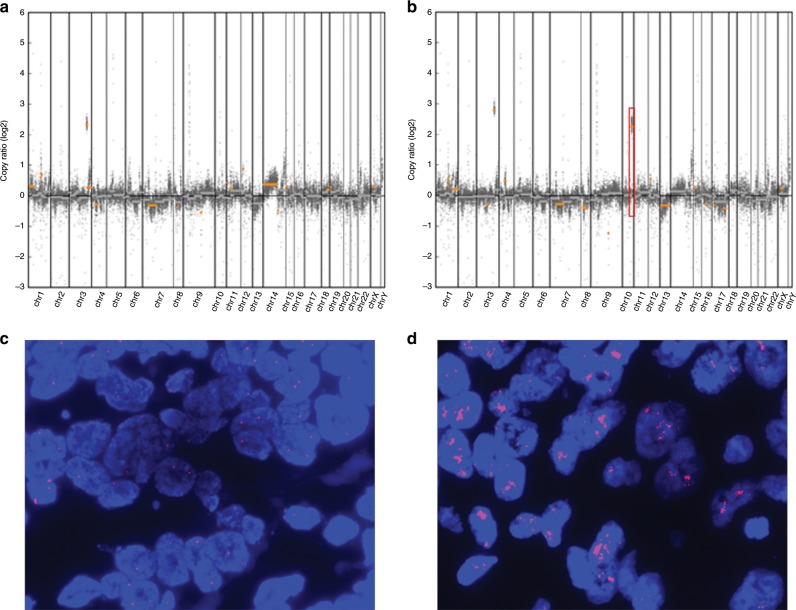
A representative case with inter-lesional copy number heterogeneity in *FGFR2* gene. Genome-wide copy number frequency plots (**a** Tumour #1, **b**: Tumour #2) and *FGFR2* florescence in situ hybridisation (FISH) images (**c** Tumour #1, **d** Tumour #2) in patient 4. *PIK3CA* amplification is detected in both tumours #1 and #2. However, *FGFR2* amplification is identified only in tumour #2 (box). Average copy number of *FGFR2* is 25.8 by FISH.

Genomic heterogeneity of copy number alteration in tumour #1 and tumour #2 was observed in 7 (33%) of 21 patients including *ERBB2* in two patients (patients 9 and 19), *FGFR1* in two patients (patients 3 and 13), and *FGFR2* in one patient (patient 4). We performed ISH to confirm the results of *ERBB2*, *FGFR1*, and *FGFR2* status in the two lesions, and we confirmed amplification in either tumour #1 or tumour #2. In an amplified tumour, the average copy number of *ERBB2* was 5.7 (with HER2/CEP17 ratio of 3.2) in patient 9 (Fig. 1), and 8.7 in patient 19. The average copy number of *FGFR1* was 9.1 in patient 3 (Fig. 2), and 16.3 in patient 13. The average copy number of *FGFR2* was 25.8 in patient 4 (Fig. 3).

Notably, one *FGFR1*-amplified case (patient 13) and one *FGFR2*-amplified case (patient 4) showed high-level amplification in the smaller tumour rather than the largest tumour. In patient 9, *SMO*, *NTRK3*, *IDH2*, *IGF1R*, and *CDK12* were also amplified in tumour #1 in addition to *ERBB2* amplification (Fig. 1). In patient 1, *CCND1* amplification was identified solely in tumour #1. In patient 10, *CCND1* and *TSC2* amplification were identified only in tumour #1. The distance between the tumour pairs showing inter-lesional copy number heterogeneity was between 0.5 and 3.5 cm (Supplementary Table S2).

## Discussion

Multifocal or multicentric breast cancers are relatively common, and they tend to share similar histology and biomarker status. However, genomic inter-lesional heterogeneity in multiple breast cancers is not well-documented. In this study, we compared genetic variations in two representative lesions with similar histologic features from 21 multiple breast cancers.

Our study showed that mutations across individual lesions from the same patient were highly concordant: it was the discordance in copy number alteration that was more frequent. Copy number status was different in 7 of 21 patients. The discordant genes included clinically relevant genes such as *ERBB2*, *FGFR1*, and *FGFR2*. HER2 overexpression and/or *ERBB2* amplification is established as the sole predictive marker for treatment benefits from HER2-targeted therapy. As for FGFR, accumulating evidence supports FGFRs as a new therapeutic target for breast cancers, especially for high-level *FGFR1*- and *FGFR2*-amplified cancers.^29,30^ However, in this study, two patients revealed high-level *FGFR* amplification in the smaller lesion. Our results indicate that performing a molecular test in the largest-index tumour could miss important molecular alterations in the smaller lesions in advanced multiple breast cancers.

Data on inter-lesional genomic heterogeneity in multiple breast cancer are limited. Yates et al. discovered the existence of complex admixture of subclones across widely separated foci in four cases of multifocal breast cancer. They observed two distinct *PTEN* driver mutations from different regions in one patient and a *CDK6* amplification localised to only one focus in another patient.^11^ Such investigation of sub-clonal structure in multifocal breast cancer showed sub-clonal growth and dissemination during tumour progression. Norton et al. investigated copy number of 80 known cancer genes in all tumour foci from 11 patients with multifocal invasive lobular breast cancer and reported that copy number status was genetically homogenous across different foci in an individual patient.^31^ Recently, Desmedt et al. conducted targeted sequencing in different lesions from 36 multifocal breast cancers.^14^ Inter-lesional heterogeneity of oncogenic mutations such as *PIK3CA*, *TP53*, *GATA3*, and *PTEN* was present in 33% of the cases. Upon copy number analysis in eight patients, inter-lesional differences in *PTEN* loss and *MYC* amplification were observed.^14^ They also investigated whether inter-lesional heterogeneity was associated with clinicopathologic factors and found that the only association that existed was with inter-lesion distance.^14^ The distance between the tumours ranged from 0.3 to 7.0 cm (mean 2.4, median 1.9) in their study.^14^ In our study, the distance between tumours was not drastically different, ranging from 0.5 to 4.5 cm (mean 1.7, median 1.0). The two tumour pairs that showed inter-lesional mutation heterogeneity tended to be further apart from each other compared to the other tumours. However, our study is limited in that the number of cases was too small to perform statistical analysis. Large-scale studies are warranted to determine clinicopathologic factors associated with inter-lesional heterogeneity.

In terms of the biology of breast cancer, there is substantial evidence that breast cancer is mainly driven by copy number alterations.^32,33^ In their study of 2000 breast tumours, Curtis et al. have observed that copy number alterations accounted for the greatest variability in gene expression.^33^ A recent investigation using a novel single-cell genome sequencing method revealed that single-cell copy number profiles were fairly similar, suggesting that this structural variation occurs early on followed by stable clonal expansions to form a tumour mass.^13^ In contrast, mutations occurring from defects in DNA repair or replication accumulate more gradually over cell divisions.^13^ Interestingly, inter-lesional difference in copy number was more prominent than point mutation in the present study. However, the number of patients was too small to draw a conclusion. A large-series study investigating copy number alterations in multiple breast cancers is warranted.

Besides genomic heterogeneity between lesions, we identified discrepancies in intrinsic subtype in 2 (10%) of 21 patients. In our study, discordance of subtypes did not affect treatment planning in those two patients. However, inter-lesional HER2 heterogeneity may be associated with poor response to HER2-targeted therapy including trastzumab, as intra-tumoural HER2 heterogeneity.^27^ Although the clinical significance of subtype discordance in multiple breast cancer has not been determined yet, recognition of inter-lesional heterogeneity of standard biomarkers such as HER2 may provide additional information on treatment response in those patients.

Our study has several limitations. First, not all copy number alteration and mutation results were validated with other methods. In situ hybridisation was performed for select genes. The discordant point mutations between tumours #1 and #2 had low variant allele frequency. We performed Sanger sequencing to confirm discordant point mutations in both tumours #1 and #2 of patients 3 and 8 but failed to confirm these mutations in both patients. It is probably due to the low sensitivity of Sanger sequencing, as the threshold for detection rests at an allele frequency of approximately 15−20%.^34^ However, we presume that these were true mutations considering there was no artefact in other genes and those were alleged as hot spot mutations. Second, instead of investigating inter-lesional heterogeneity thoroughly from all lesions in an individual patient, we selected only two largest lesions. Lastly, no alterations were detected in four patients but the tumour purity of those cases was between 70 and 90%. Thus, it was probably due to the limitations pertaining to the targeted gene panel. The number of genes covered in the present study was relatively small. Of the 170 genes in our panel, 134 genes overlapped with those from Desmedt et al.’s panel.^14^ Furthermore, genome-wide comparison between lesions was not available with the targeted gene panel. In further studies, genomic investigation of multiple invasive cancers and the associated carcinoma in situ in the whole genome level may provide insight into clonal evolution and timing of divergence in multiple breast cancers. Prospective integration of genomic studies and clinical trials is also warranted.^11,35^

In conclusion, inter-lesional genomic heterogeneity, particularly of copy number alteration, was identified in a substantial number of multiple breast cancers. In precision medicine, inter-lesional genomic heterogeneity should be considered in representative tumour sampling and molecular testing in multiple breast cancers.

## Supplementary information

Supplementary tables

## Data Availability

All data and materials are available by inquiring to the corresponding author.

## References

[1] Boyages J, Jayasinghe UW, Coombs N (2010). Multifocal breast cancer and survival: each focus does matter particularly for larger tumours. Eur. J. Cancer.

[2] Buggi F, Folli S, Curcio A, Casadei-Giunchi D, Rocca A, Pietri E (2012). Multicentric/multifocal breast cancer with a single histotype: is the biological characterization of all individual foci justified?. Ann. Oncol..

[3] Lynch SP, Lei X, Chavez-MacGregor M, Hsu L, Meric-Bernstam F, Buchholz TA (2012). Multifocality and multicentricity in breast cancer and survival outcomes. Ann. Oncol..

[4] Neri A, Marrelli D, Megha T, Bettarini F, Tacchini D, De Franco L (2015). "Clinical significance of multifocal and multicentric breast cancers and choice of surgical treatment: a retrospective study on a series of 1158 cases". BMC Surg..

[5] Fisher ER, Gregorio R, Redmond C, Vellios F, Sommers SC, Fisher B (1975). Pathologic findings from the National Surgical Adjuvant Breast Project (protocol no. 4). I. Observations concerning the multicentricity of mammary cancer. Cancer.

[6] Amin M. B., Edge S., Greene F. L., Byrd D. R., Brookland R. K., Washington M. K. et al. *AJCC Cancer Staging Manual.* 8th edn. (Springer Nature: Basel, 2017).

[7] Middleton LP, Vlastos G, Mirza NQ, Eva S, Sahin AA (2002). Multicentric mammary carcinoma: evidence of monoclonal proliferation. Cancer.

[8] Choi Y, Kim EJ, Seol H, Lee HE, Jang MJ, Kim SM (2012). The hormone receptor, human epidermal growth factor receptor 2, and molecular subtype status of individual tumor foci in multifocal/multicentric invasive ductal carcinoma of breast. Hum. Pathol..

[9] Lester SC, Bose S, Chen YY, Connolly JL, de Baca ME, Fitzgibbons PL (2009). Protocol for the examination of specimens from patients with invasive carcinoma of the breast. Arch. Pathol. Lab. Med..

[10] College of American Pathologists. Protocol for the examination of specimens from patients with invasive carcinoma of the breast. https://cap.objects.frb.io/protocols/public-comment-drafts/cp-breast-invasive-biopsy-19-1000-draftPC.pdf. (2019).

[11] Yates LR, Gerstung M, Knappskog S, Desmedt C, Gundem G, Van Loo P (2015). Subclonal diversification of primary breast cancer revealed by multiregion sequencing. Nat. Med..

[12] Murtaza M, Dawson SJ, Pogrebniak K, Rueda OM, Provenzano E, Grant J (2015). Multifocal clonal evolution characterized using circulating tumour DNA in a case of metastatic breast cancer. Nat. Commun..

[13] Wang Y, Waters J, Leung ML, Unruh A, Roh W, Shi X (2014). Clonal evolution in breast cancer revealed by single nucleus genome sequencing. Nature.

[14] Desmedt C, Fumagalli D, Pietri E, Zoppoli G, Brown D, Nik-Zainal S (2015). Uncovering the genomic heterogeneity of multifocal breast cancer. J. Pathol..

[15] Yates LR (2017). Intratumoral heterogeneity and subclonal diversification of early breast cancer. Breast.

[16] Goldhirsch A, Wood WC, Coates AS, Gelber RD, Thurlimann B, Senn HJ (2011). Strategies for subtypes–dealing with the diversity of breast cancer: highlights of the St. Gallen International Expert Consensus on the Primary Therapy of Early Breast Cancer 2011. Ann. Oncol..

[17] Martin M (2011). Cutadapt removes adapter sequences from high-throughput sequencing reads. EMBnetjournal.

[18] Li, H. Aligning sequence reads, clone sequences and assembly contigs with BWA-MEM. arXiv.1303.3997v2 (2013).

[19] Li H, Handsaker B, Wysoker A, Fennell T, Ruan J, Homer N (2009). The Sequence Alignment/Map format and SAMtools. Bioinformatics.

[20] Cibulskis K, Lawrence MS, Carter SL, Sivachenko A, Jaffe D, Sougnez C (2013). Sensitive detection of somatic point mutations in impure and heterogeneous cancer samples. Nat. Biotechnol..

[21] Cingolani P, Patel VM, Coon M, Nguyen T, Land SJ, Ruden DM (2012). Using Drosophila melanogaster as a model for genotoxic chemical mutational studies with a new program, SnpSift. Front. Genet..

[22] Liu X, Wu C, Li C, Boerwinkle E (2016). dbNSFP v3.0: a one-stop database of functional predictions and annotations for human nonsynonymous and splice-site SNVs. Hum. Mutat..

[23] Lek M, Karczewski KJ, Minikel EV, Samocha KE, Banks E, Fennell T (2016). Analysis of protein-coding genetic variation in 60,706 humans. Nature.

[24] Landrum MJ, Lee JM, Benson M, Brown G, Chao C, Chitipiralla S (2016). ClinVar: public archive of interpretations of clinically relevant variants. Nucleic Acids Res..

[25] Forbes SA, Tang G, Bindal N, Bamford S, Dawson E, Cole C (2010). COSMIC (the Catalogue of Somatic Mutations in Cancer): a resource to investigate acquired mutations in human cancer. Nucleic Acids Res..

[26] Talevich E, Shain AH, Botton T, Bastian BC (2016). CNVkit: genome-wide copy number detection and visualization from targeted DNA sequencing. PLoS Comput. Biol..

[27] Lee HJ, Seo AN, Kim EJ, Jang MH, Suh KJ, Ryu HS (2014). HER2 heterogeneity affects trastuzumab responses and survival in patients with HER2-positive metastatic breast cancer. Am. J. Clin. Pathol..

[28] Wolff AC, Hammond ME, Hicks DG, Dowsett M, McShane LM, Allison KH (2013). Recommendations for human epidermal growth factor receptor 2 testing in breast cancer: American Society of Clinical Oncology/College of American Pathologists clinical practice guideline update. J. Clin. Oncol..

[29] Perez-Garcia J, Munoz-Couselo E, Soberino J, Racca F, Cortes J (2018). Targeting FGFR pathway in breast cancer. Breast.

[30] Pearson A, Smyth E, Babina IS, Herrera-Abreu MT, Tarazona N, Peckitt C (2016). High-level clonal FGFR amplification and response to FGFR inhibition in a translational clinical trial. Cancer Discov..

[31] Norton N, Advani PP, Serie DJ, Geiger XJ, Necela BM, Axenfeld BC (2016). Assessment of tumor heterogeneity, as evidenced by gene expression profiles, pathway activation, and gene copy number, in patients with multifocal invasive lobular breast tumors. PLoS ONE.

[32] Ciriello G, Miller ML, Aksoy BA, Senbabaoglu Y, Schultz N, Sander C (2013). Emerging landscape of oncogenic signatures across human cancers. Nat. Genet..

[33] Curtis C, Shah SP, Chin S-F, Turashvili G, Rueda OM, Dunning MJ (2012). The genomic and transcriptomic architecture of 2,000 breast tumours reveals novel subgroups. Nature.

[34] Tsiatis AC, Norris-Kirby A, Rich RG, Hafez MJ, Gocke CD, Eshleman JR (2010). Comparison of Sanger sequencing, pyrosequencing, and melting curve analysis for the detection of KRAS mutations: diagnostic and clinical implications. J. Mol. Diagn..

[35] Yuan Y, Van Allen EM, Omberg L, Wagle N, Amin-Mansour A, Sokolov A (2014). Assessing the clinical utility of cancer genomic and proteomic data across tumor types. Nat. Biotechnol..

