# Supplementary material for: Genomic profiling of multiple breast cancer reveals inter-lesional heterogeneity
Source: Br J Cancer. 2020 Jan 13;122(5):697–704. doi: 10.1038/s41416-019-0713-1 (PMC7054255; doi:10.1038/s41416-019-0713-1)
Supplement: Supplementary file 1 — Supplementary tables [file 41416_2019_713_MOESM1_ESM.docx]

**Supplementary Table S1**. List of 170 cancer-related genes in targeted DNA sequencing

| ABL1 | BCL2 | CDKN1B | ERBB3 | FLCN | JAK3 | MEN1 | NOTCH3 | PPARG | SMAD4 |
| --- | --- | --- | --- | --- | --- | --- | --- | --- | --- |
| ABL2 | BRAF | CDKN2A | ERBB4 | FLT1 | KDR | MET | NOTCH4 | PTCH1 | SMARCA4 |
| AKT1 | BRCA1 | CDKN2B | ERCC2 | FLT3 | KIT | MITF | NPM1 | PTEN | SMARCB1 |
| AKT2 | BRCA2 | CDKN2C | ERG | FLT4 | KMT2A | MLH1 | NRAS | RAB35 | SMO |
| AKT3 | BRD2 | CEBPA | ERRFI1 | FOXL2 | KRAS | MPL | NTRK1 | RAD50 | SRC |
| ALK | BRD3 | CHEK2 | ESR1 | GNA11 | MAP2K1 | MSH2 | NTRK2 | RAF1 | STK11 |
| APC | BRD4 | CREBBP | ETV1 | GNAQ | MAP2K2 | MSH6 | NTRK3 | RARA | SYK |
| AR | CBFB | CRKL | ETV4 | GNAS | MAP2K4 | MTOR | NUTM1 | RB1 | TET2 |
| ARAF | CCND1 | CSF1R | ETV5 | HDAC9 | MAP3K1 | MYC | PDGFB | RET | TMPRSS2 |
| ASXL1 | CCND2 | CTNNB1 | ETV6 | HGF | MAP3K4 | MYCN | PDGFRA | RHEB | TOP2A |
| ATM | CCND3 | DDR1 | EWSR1 | HRAS | MAPK1 | MYD88 | PDGFRB | RICTOR | TP53 |
| ATR | CCNE1 | DDR2 | EZH2 | IDH1 | MAPK3 | NF1 | PIK3CA | RNF43 | TSC1 |
| AURKA | CDH1 | DNMT3A | FBXW7 | IDH2 | MAPK8 | NF2 | PIK3CB | ROS1 | TSC2 |
| AURKB | CDK12 | DOT1L | FGFR1 | IGF1R | MCL1 | NFKBIA | PIK3CD | RSPO1 | VHL |
| AURKC | CDK4 | EGFR | FGFR2 | IGF2 | MDM2 | NKX2-1 | PIK3R1 | RSPO2 | WT1 |
| AXL | CDK6 | EPHA3 | FGFR3 | JAK1 | MDM4 | NOTCH1 | PIK3R2 | RUNX1 | XPO1 |
| BAP1 | CDKN1A | ERBB2 | FGFR4 | JAK2 | MED12 | NOTCH2 | POLE | SMAD2 | ZNRF3 |

**Supplementary Table S2**. Characteristics of tumors and SNV/CNV heterogeneity

| Patient | Total number of lesions | Size of the largest lesion (cm) | pN stage | Distance between lesions (cm) | Histologic grade | Subtype | |  | Tumor purity (%) | | SNV/CNV | SNV heterogeneity | CNV heterogeneity |
| --- | --- | --- | --- | --- | --- | --- | --- | --- | --- | --- | --- | --- | --- |
|  |  |  |  |  |  | Tumor 1 | Tumor 2 |  | Tumor 1 | Tumor 2 |  |  |  |
| 1 | ≥ 3 | 3 | N1 | 1.0 | 3 | Luminal B/HER2 negative | Luminal B/HER2 negative |  | 70 | 70 | Detected | Absent | Present |
| 2 | 2 | 5.5 | N0 | 1.0 | 3 | Luminal B/HER2 negative | Luminal B/HER2 negative |  | 80 | 90 | Not detected | Absent | Absent |
| 3 | 2 | 1.6 | N0 | 3.5 | 2 | Luminal A | Luminal A |  | 80 | 80 | Detected | Present | Present |
| 4 | ≥ 3 | 2.2 | N2 | 0.5 | 3 | Luminal B/HER2 negative | Luminal B/HER2 negative |  | 70 | 80 | Detected | Absent | Present |
| 5 | ≥ 3 | 5.5 | N3 | 2.5 | 3 | Luminal A | Luminal A |  | 70 | 80 | Detected | Absent | Absent |
| 6 | 2 | 2.1 | N1 | 1.0 | 2 | Luminal A | Luminal A |  | 80 | 90 | Not detected | Absent | Absent |
| 7 | ≥ 3 | 2 | N1 | 2.5 | 3 | Luminal B/HER2 negative | Luminal B/HER2 negative |  | 80 | 80 | Detected | Absent | Absent |
| 8 | 2 | 2.4 | N1 | 4.0 | 3 | HER2 positive | HER2 positive |  | 80 | 80 | Detected | Present | Absent |
| 9 | ≥ 3 | 1.8 | N0 | 2.0 | 3 | Luminal B/HER2 positive | Luminal B/HER2 negative |  | 70 | 70 | Detected | Absent | Present |
| 10 | 2 | 1.4 | N0 | 0.5 | 3 | Luminal B/HER2 positive | Luminal B/HER2 positive |  | 70 | 70 | Detected | Absent | Present |
| 11 | 2 | 1.4 | N0 | 0.5 | 2 | Luminal A | Luminal A |  | 80 | 80 | Not detected | Absent | Absent |
| 12 | 2 | 1.5 | N1 | 0.5 | 2 | Luminal B/HER2 negative | Luminal B/HER2 negative |  | 70 | 70 | Detected | Absent | Absent |
| 13 | 2 | 1.5 | N0 | 2.5 | 3 | HER2 positive | HER2 positive |  | 50 | 60 | Detected | Absent | Present |
| 14 | ≥ 3 | 2.9 | N1 | 0.5 | 2 | Luminal A | Luminal A |  | 70 | 70 | Not detected | Absent | Absent |
| 15 | 2 | 1.6 | N0 | 0.5 | 3 | HER2 positive | HER2 positive |  | 70 | 70 | Detected | Absent | Absent |
| 16 | 2 | 2.6 | N0 | 1.0 | 2 | Triple-Negative | Triple-Negative |  | 70 | 70 | Detected | Absent | Absent |
| 17 | ≥ 3 | 4.8 | N0 | 0.5 | 3 | HER2 positive | HER2 positive |  | 80 | 80 | Detected | Absent | Absent |
| 18 | ≥ 3 | 3.8 | N3 | 4.5 | 3 | Luminal B/HER2 positive | Luminal B/HER2 positive |  | 70 | 70 | Detected | Absent | Absent |
| 19 | 2 | 1.7 | N0 | 2.0 | 3 | HER2 positive | Triple-Negative |  | 80 | 80 | Detected | Absent | Present |
| 20 | 2 | 2.2 | N0 | 1.5 | 3 | Luminal B/HER2 negative | Luminal B/HER2 negative |  | 50 | 50 | Detected | Absent | Absent |
| 21 | 2 | 1.8 | N0 | 3.0 | 1 | Luminal B/HER2 negative | Luminal B/HER2 negative |  | 70 | 80 | Detected | Absent | Absent |
| SNV, single nucleotide variant; CNV, copy number variant | | | | | | | |  |  |  |  |  |  |
|  | | | | | | | |  |  |  |  |  |  |
